# How does the situation before a tackle influence a tackler’s head placement in rugby union?: application of the decision tree analysis

**DOI:** 10.1136/bmjsem-2020-000949

**Published:** 2021-03-17

**Authors:** Keita Suzuki, Satoshi Nagai, Koichi Iwai, Takuo Furukawa, Masahiro Takemura

**Affiliations:** 1 Sports Research and Development Core, University of Tsukuba, Tsukuba, Japan; 2 Department of Physical Therapy, Faculty of Health Sciences, Tsukuba International University, Tsuchiura, Ibaraki, Japan; 3 Center of Humanities and Sciences, Ibaraki Prefectural University of Health Sciences, Inashiki-gun, Ibaraki, Japan; 4 Faculty of Health and Sport Sciences, University of Tsukuba, Tsukuba, Ibaraki, Japan

**Keywords:** rugby, university, sporting injuries, concussion

## Abstract

**Objectives:**

Tacklers need to decide where to place their head based on the evasive manoeuvres of the ball-carrier and positional relationship with the ball-carrier before tackle. Therefore, it is difficult for tacklers to improve incorrect head placement at the moment of contact. Moreover, the characteristics prior to tackle have a relationship with the tackler’s head placement. However, how situations lead to incorrect head placement remains unknown. The aim of this study was to identify pre-contact situations that lead to incorrect head placement by using decision tree analysis.

**Methods:**

Tackles leading to concussions were used to identify events that provoked injury using the video recordings of matches. Injury-free tackle was used as a control. All tackles were classified according to head placements and coded from seven pre-contact factors configured aspect of both tacklers and ball-carriers.

**Results:**

Three situations that led to incorrect head placement were identified. Evasive manoeuvres implemented by the ball-carrier significantly contributed to the head placement at the time of contact.

**Conclusion:**

Our findings suggest that tacklers should keep their heads up to identify the movements of the ball-carrier, which might lead to tackling the head on the correct side at the moment of tackling and decrease the risk of tackler-related concussions.

**What are the new findings?**

- The probability of tackler-related concussions increased when tackles were attempted with incorrect head placement.
- Three situations before contact were associated with a greater frequency of tackles with incorrect head placement.
- Evasive manoeuvres by the ball-carrier also contributed to the head placement at the time of contact.
- The frequency of the tackle with a correct head placement increased when the tackler faced the ball-carrier or the tackler’s head moved in an attempt to follow the ball-carrier.

## Introduction

**Figure 1:**
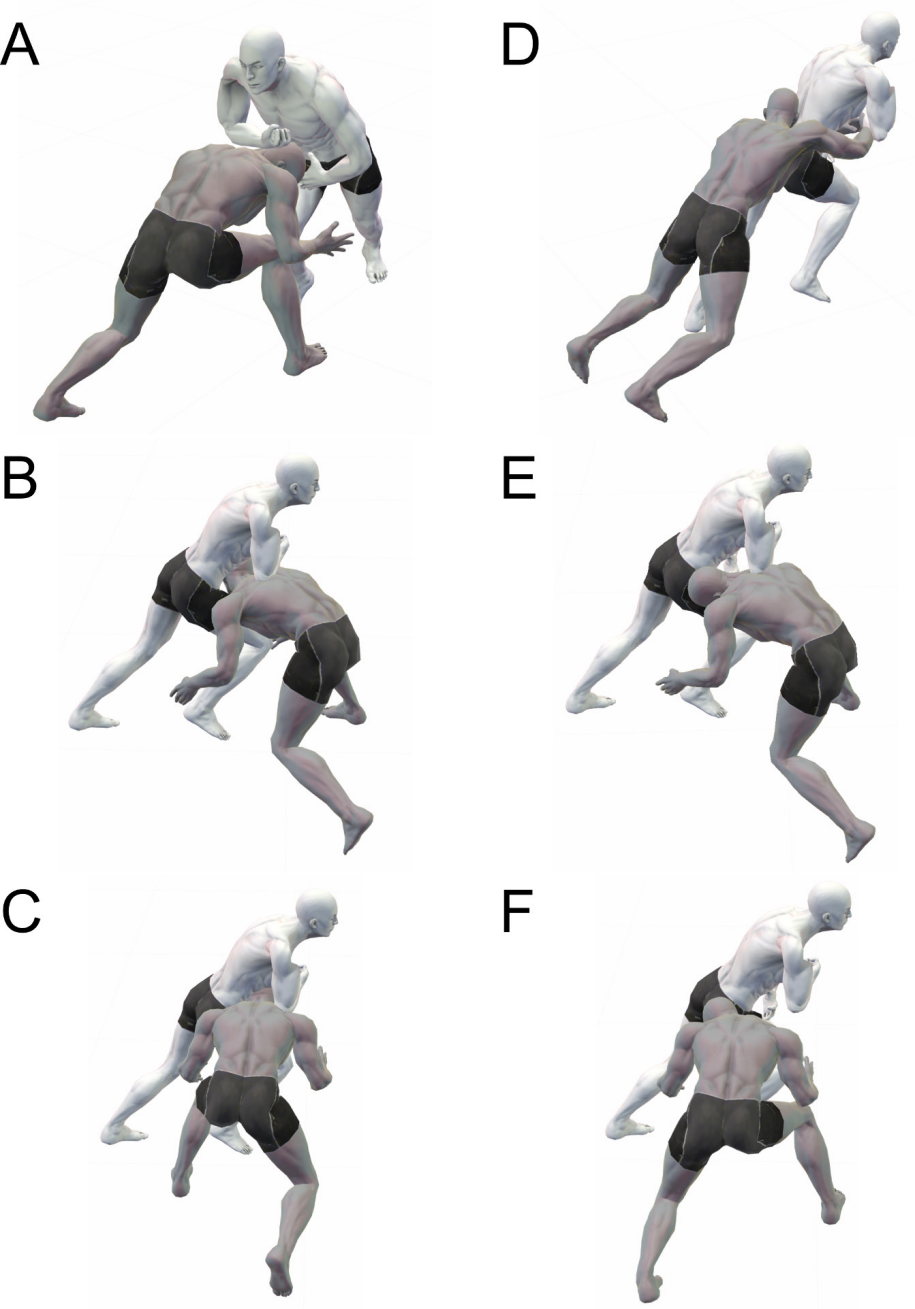
Definition of the incorrect tackler’s head placement. (A) The tackler’s head is in front of the ball-carrier during a front-on tackle. (B) The tackler’s head is in front of the ball-carrier during a tackle from oblique. (C) The tackler’s head is in front of the ball-carrier during a side-on tackle. (D) The tackler’s head is behind the ball-carrier during a tackle from behind. (E) The tackler’s head is placed at one side of the ball-carrier during a tackle from oblique. (F) The tackler’s head is placed at one side of the ball-carrier during a side-on tackle. * Grey, a tackler; white, a ball-carrier.

Video analysis of tackle-related injuries,^1–5^ including concussions,^6–14^ neck injuries^15^ and shoulder injuries,^16^ has been conducted in rugby players at various competitive levels. In addition, head impact tackle scenes^17 18^ and tackle performance^19 20^ have been analysed using video footage. The risk of tackle-related injuries tends to increase when the tackler’s head is in front of the ball-carrier.^3 7–9 15 16^ In addition, our previous study showed that the likelihood of concussion increased when the head of the tackler was placed at one side of the ball-carrier.^8^ For a side-on or oblique tackle, the tackler’s head on the side of the ball-carrier may be not the correct head position; however, for a front-on tackle, the tackler’s head on the side of the ball-carrier is correct. In other words, it may not be sufficient to classify the tackler’s head placement with respect to the ball-carrier. Sobue *et al* defined an incorrect head placement as that with the tackler’s head in front of the ball-carrier, which has been mentioned as a risk for the occurrence of concussions.^21^ However, the effect of other head placements (lateral or posterior to the ball-carrier) on the occurrence of the tackler-related concussion have not been clarified. Therefore, in the present study, an incorrect head placement was defined as satisfying either (1) when the tackler’s head was placed in front of the ball-carrier (figure 1A–C)^21^ or (2) when any tackles gave an impact force to the tackler’s head from the diagonal, lateral or posterior direction (figure 1D–F).

The characteristics of both the tackler and ball-carrier prior to tackle are associated with the occurrence of tackler-related concussion. Regarding the tackler, whether the tackler is accelerating,^6 13^ looking forward with face raised,^8 9^ recognising the ball-carrier,^9^ shortening steps^9 14^ and the tackler’s posture before the tackle^13^ are related to the occurrence of tackler-related concussion. In the aspect of the ball-carrier, an accelerating ball-carrier^6 13^ and evasive manoeuvre prior to tackle^8^ have a relationship with the occurrence of tackler-related concussion. In addition, the latest systematic scoping review reported that ‘head up and facing forward’ and ‘shortening steps’ were associated with injury prevention and tackling performance in rugby union.^22^ Therefore, tacklers need to decide where to place their own head based on the evasive manoeuvres of the ball-carrier and positional relationship with the ball-carrier before tackle. Thus, it is difficult for tacklers to improve incorrect head placement at the moment of contact. In other words, situations prior to tackling might have a relationship with the tackler’s head placement, which is a risk of tackle-related injuries, including concussions. However, coaches often focus attention on the contact phase during tackle-technique training while excluding the pre-contact phase.^23^

It is necessary to identify the situations leading to the main outcome in order to clarify the relationship between the combination of multiple factors and the main outcome. In addition, we believe that it is important that the results of research be easily understood by coaches and players in order to apply them in their coaching and training. Therefore, we focused on decision tree analysis, a machine learning technique that has recently been used in the field of sports science. Decision tree analysis, a machine learning technique, has been used to explain certain situations in one-versus-one player interactions,^24^ match outcomes,^25–28^ and the effectiveness of ball-screens and inside-passes in basketball.^29 30^ Therefore, applying decision tree analysis could reveal pre-contact situations involving the tackler’s head placement. The present study aimed to identify the pre-contact situations that lead to incorrect head placement by using decision tree analysis.

## Methods

### Concussion data

The present study was a case–control study. Injury surveillance of a Japanese collegiate rugby union team was conducted between 2008 and 2015 with informed consent from the players.^8^ There was no patient or public involvement.

In all playing seasons, medically trained personnel from teams recorded injuries according to the consensus statement for injury surveillance.^31^ Furthermore, all players who sustained a head impact or who sustained a suspected concussion during matches were screened by a team doctor in accordance with the Concussion Guidance published by the World Rugby.^32^ All suspected or confirmed concussions were analysed in the present study.^33^

### Tackle data

A tackle has been defined as any event in which one or more tacklers attempt to stop or impede the ball-carrier, whether or not the ball-carrier was brought to the ground.^3^ We used data from our previous study that collected tackles leading and not leading to concussions.^8^ These data included tackles from 34 collegiate rugby union team matches recorded between 2008 and 2015, in which the tacklers sustained concussions. We selected 538 tackles, 34 of which led to concussions (experimental group) and 504 did not result in any type of injury (control group). An a priori power analysis indicated that an estimated total sample size of 208 events, including both concussion and non-injury events, would be required to achieve a power (1−β) of 0.80 with a confidence level of 95% (α=0.05), while an OR of 1.50 is required to produce the smallest worthwhile effect.

### Data analysis

We defined an incorrect head placement as satisfying either (1) when the tackler’s head was in front of the ball-carrier (figure 1A–C)^21^ or (2) when any tackles gave an impact force to the tackler’s head contacting from the diagonal, lateral or posterior direction (figure 1D–F). The first author (KS) coded all tackles described previously from seven factors, which configured the aspect of both the tackler and ball-carrier during the pre-contact phase based on both consensus statement on video analysis in rugby union and previous studies (table 1).^8 19 34–36^ Tackles were excluded when the placement of the tackler’s head at the time of contact could not be identified. According to our previous research, we obtained high inter-rater reliability between the two raters.^37^ The κ statistic result for inter-tester reliability was 0.85 (pre-contact), indicating excellent agreement.^38^

**Table 1:** Characteristics of pre-contact phase variable and their descriptions

Categorical variables	Descriptions
*Tackler*	
Stance^19^	
Flat footed	Tackler standing square with feet aligned and flat on the ground
Back foot	Tackler stepping backwards as ball-carrier approaches
Split forward	Tackler standing with staggered stance
No stance	Tackler diving or sliding into contact
Direction of movement of tackler^8 19^	
Forward	Toward the ball-carrier
Backwards	Back pedalling, that is, away a ball-carrier
Lateral	Towards the touchline (across the field)
No movement	Tackler did not move before tackle situation
Head position^19 36^	
Up and forward	Towards ball-carrier
Away	Away from ball-carrier
Down	Towards the ground
In motion	Tackler’s head was moving in attempt to follow the ball-carrier
Speed of tackler^34 36^	
Fast	Running/sprinting (purposeful running with maximal effort, high knee lift)
Moderate	Jogging (non-purposeful slow running with low knee lift)
Slow	Stationary/walking (no or few visible foot movement)
Orientation of tackler in relation to ball-carrier^19^	
In front	Tackler and ball-carrier moving head on towards each other
Side	Tackler moving in from the ball-carrier’s side
Oblique	Tackler moving into ball-carrier at an angle
Behind	Tackler chasing ball-carrier towards own try-line
*Ball-carrier*	
Speed of ball-carrier^34 36^	
Fast	Running/sprinting (purposeful running with maximal effort, high knee lift)
Moderate	Jogging (non-purposeful slow running with low knee lift)
Slow	Stationary/walking (no or few visible foot movement)
Evasive manoeuvre performed by ball-carrier^8 35 36^	
Straight run	Ball-carrier running straight at the defence or goal-line
Side-step	Ball-carrier performing an evasive step initiated by either leg
Arcing run	Ball-carrier performing arcing run
Lateral run	Ball-carrier performing a run from touchline to touchline
Diagonal run	Ball-carrier running at an angle, instead of straight at the tackler
Be tackled	Ball-carrier being tackled by the other tacklers and no movement in any direction
No movement	Ball-carrier not moving before tackle situation

### Statistical analysis

When we used the tackler’s head placement as the main outcome of the decision tree analysis, we needed to clarify whether it is also related to concussion in this cohort. Therefore, we calculated OR and 95% CIs from the results of the binomial logistic regression analysis. If the OR was >1.0, concussion was more likely to occur compared with correct head placement. We also recorded situations in pre-contact features that led to incorrect head placement using the decision tree within the *rpart* package^39^ using *R* V.3.5.3 (R Foundation for Statistical Computing, Vienna, Austria). The *rpart* package uses the classification and regression tree (CART) algorithm to build a tree model. In CART, some variables merge such that there are always two branches based on the Gini indexes.

### Patient and public involvement

Patients and/or the public were not involved in the design, or conduct, or reporting, or dissemination plans of this research.

## Results

After excluding 49 tackles that could not be identified with the placement of the tackler’s head at the time of contact, we analysed data from 489 tackles (90.9%), including 34 that led to a concussion (100%) and 455 that did not result in any type of injury (90.2%). Of these, we classified head placements in 140 and 349 tackles as incorrect and correct, respectively.

### Effects of incorrect head placement on the risk of concussion

First, we confirmed whether the tackler’s head placement was related to concussion by logistic regression analysis in this cohort, in order to use that binary variable as the main outcome of the decision tree analysis. The risk of concussion was significantly higher in tackles with incorrect head placement (OR 8.21; 95% CI 3.73 to 18.11; p<0.01; table 2). In addition, we assigned tackles with incorrect head placements to groups depending on when the tackler’s head was in front of the ball-carrier or when any tackles gave an impact force to the tackler’s head contacting from the diagonal, lateral or posterior direction. The risk of concussion increased when the tackler’s head was in front (OR 14.78; 95% CI 6.27 to 34.84; p<0.01; table 2), and when any tackles gave impact force to the tackler’s head contacting from the diagonal, lateral or posterior direction (OR 3.83; 95% CI 1.38 to 10.64; p=0.01; table 2) with the ball-carrier compared with the correct head placement.

**Table 2:** Tackler head placements during contact are associated with the tackler-related concussion occurrence

Head placements (vs no injuries)	OR	(95% CI)	P value
Correct	Reference		
Incorrect*	8.21	(3.73 to 18.11)	<0.01
Correct	Reference		
Incorrect (1)†	14.78	(6.27 to 34.84)	<0.01
Incorrect (2)‡	3.83	(1.38 to 10.64)	0.01

*An incorrect head placement as satisfying either (1) when the tackler’s head was in front of the ball-carrier or (2) when any tackles gave an impact force to the tackler’s head in the diagonal, lateral or posterior direction.

†An incorrect head placement as satisfying (1) when the tackler’s head was in front of the ball-carrier.

‡An incorrect head placement as satisfying (2) when any tackles gave an impact force to the tackler’s head contacting from the diagonal, lateral or posterior direction.

CI, confidence intervals; OR, odds ratio.

### Situations before contact leading to tackles with incorrect head placement

**Figure 2:**
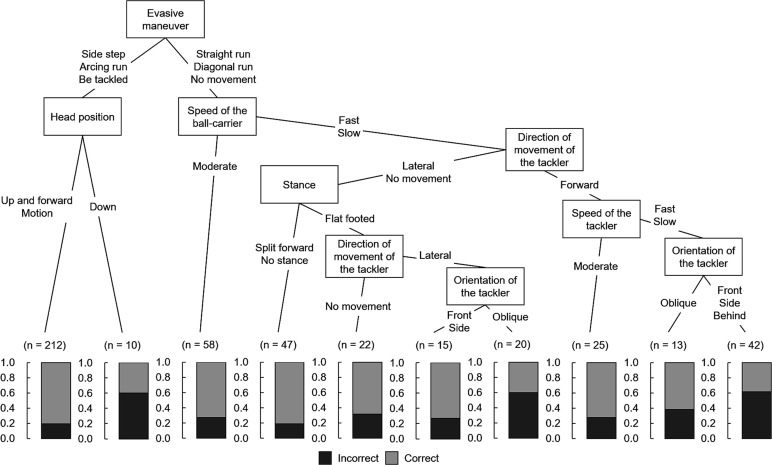
Decision tree according to tackler head placements.

Figure 2 shows the features associated with a higher probability of tackling an incorrect head placement at the time of contact. The frequency of tackles with an incorrect head placement was 60.0% when a ball-carrier side-stepped, ran an arc or was tackled by others, and when the tackler’s head was angled downward.

The frequency of an incorrect head placement was also 60.0% when ball-carriers ran straight or diagonally towards the defence line, when they did not move, or moved at high or low speed; and when the tackler moved laterally, stood with both feet flat on the ground or was located obliquely to the ball-carrier.

Evasive manoeuvres and speed of the ball-carrier were the same as the second set of features. Furthermore, the frequency of tackles with an incorrect head placement was 61.9% when the tacklers moved forward, moved at high or low speed, and were located in front of, next to or behind the ball-carrier.

## Discussion

This is the first attempt to identify the situations before contact leading to tackles with incorrect head placement by using decision tree analysis. To the best of our knowledge, no research on tackle has applied decision tree analysis in the rugby union. Thus, the present study is the first to present pre-contact situations, which configured the aspect of both tackler and ball-carrier, related to the tackler’s head placement.

### Relationship between incorrect head placement and concussion

The risk of tackler-related concussion increased 8.2-fold with tackles with incorrect head placement, which concurred with some previous findings.^7–9 21^ In addition to the traditional definition of incorrect head placement, when the tackler’s head was in front of the ball-carrier, we also analysed an incorrect head placement when a tackler’s head was in a position of contact with other players without in front of the ball-carrier. As a result, the risk of concussion was significantly increased (OR 3.83) even after excluding tackles with the head in front of the ball-carrier. Rugby Ready, which is a website intended to raise awareness of good practice and help stakeholders manage the inherent risks of contact by the World Rugby, advises positioning the head behind or to one side, and never in front of the ball-carrier in all tackle situations.^40^ Our results provide support for this recommendation by demonstrating the combined effects of head placement of the tackler and direction of the tackle.

### Situations leading to the placement of the tackler’s head at the time of contact

Evasive manoeuvres implemented by the ball-carrier significantly contributed to the head placement at the time of contact. The probability of tackles with incorrect head placement also increases when a ball-carrier tries to avoid the tackle by side-stepping and running an arc, or when the tackler’s head faces downward. Conversely, even if the evasive manoeuvre of the ball-carrier was the same, the frequency of the tackle with a correct head placement increased when the tackler faced toward the ball-carrier or tackler’s head was moving in an attempt to follow the ball-carrier. At a professional level, the characteristics ‘head up and forward/face up’ and ‘identify/track ball-carrier onto shoulder’ had a lower propensity to result in a head injury assessment for tacklers.^9^ Further, lowered tackler’s head increased the frequency of tackler’s primary anterior shoulder dislocation.^16^ Moreover, in terms of tackle performance, having the head up, looking forward and facing the ball-carrier before contact affected successful outcomes.^19 20^ Therefore, our results suggest that tacklers should keep their heads up to identify the movements of the ball-carrier, which might lead to tackling the head on the correct side at the moment of tackling and decrease the likelihood of tackler-related injuries and tackle failure.

In addition, when the ball-carrier ran straight to the defence line at high speed, the likelihood of tackling an incorrect head placement increased whether a tackler moved at high or low speeds. When both the tackler and the ball-carrier sprinted, the estimated contact time was shortened, which would interfere with the ability of the tacklers to control their own movements at the time of contact. Conversely, the risk of tackles with incorrect head placements decreased in the same situation mentioned previously, even when a tackler moved at a moderate speed. Therefore, these results suggest that controlling the speed of tacklers to match the movements of ball-carriers is essential. For example, shortening steps to control speed might help to prevent tacklers from placing incorrect head positions and improve tackle performance. ‘Shortening steps’ was significant in enabling successful tackles^20^ and decreasing the likelihood tackle-related injuries.^2 14^

In the present study, we suggested that the two aforementioned characteristics, ‘head up’ and ‘controlling the tackler’s speed’, decrease the probability of incorrect head placement during the tackle. We applied the decision tree analysis for clarifying the situations that led to tackler’s head placement. As a result, we identified the situations that led to both tackling correct head placement and incorrect head placement. We believe that analysis of the situations that decreased the risk of concussion might lead to better recommendations for the prevention of concussion.

### Applying decision tree analysis

In the present study, we clarified the relationship between a tackler’s head placement (the main outcome) and the pre-contact situations of a tackler and the ball-carrier by decision tree analysis. We believe that the decision tree analysis is an approach that allows players, coaches and trainers to visually understand the relationship between the main outcome and multiple factors. Our results showed that the probability of tackles with correct head placement increased regardless of the direction of the tackle when a ball-carrier tries to avoid the tackle by side-stepping, running an arc, and when a tackler faced toward the ball-carrier or tackler’s head was moving in an attempt to follow the ball-carrier. When players, especially beginners, improve their interpersonal tackle technique by restricting the ball-carrier’s movement and keeping the tackler’s head up to check the ball-carrier’s movements, they will contribute to training under safe conditions with a lower probability of concussions.

Moreover, this is the first study to adapt decision tree analysis to video analysis of tackling scenes in rugby union. The results showed that a combination of pre-contact characteristics influenced the head placement of the tackler. Therefore, we believe that new insights can be identified by adapting decision tree analysis to other tackle-related injury mechanisms and tackle performance.

### Limitations

The present study generated important information, and several limitations should be addressed. At first, our study analysed isolated characteristics in the pre-contact phase, which were not involved in any other players or the ground area occurred a tackle. As concussions occur after contact, our results do not directly influence the occurrence of concussions. Therefore, future studies need to determine the effects of concussion by combining the phases in which concussions occur with some of the situations identified in this study. Second, since our results were based on a single collegiate rugby union team, caution should be exercised when generalising our findings, even at the same playing level. Other contextual factors, such as match time and match status at the time of tackles, were not analysed. Tierney *et al* reported that most direct impacts to the heads of tacklers occur during the second half of matches.^18^ Therefore, the characteristics of tackles with incorrect head placements might differ significantly according to the amount of time spent in matches.

## Conclusions

We demonstrated the effects of incorrect tackler head placements and the direction of tackles on the risk of tackler-related concussion and identified three situations before contact, which led to tackling incorrect head placement. The risk of tackler-related concussions increased when tackles were attempted with incorrect head placement. Evasive manoeuvres by the ball-carrier also contributed to the head placement at the time of contact. Therefore, our results suggest that tacklers should keep their heads up to identify the movements of the ball-carrier, which might lead to tackling the head on the correct side at the moment of tackling and decreasing the risk of tackler-related concussion.
